# Midbrain atrophy related to parkinsonism in a non-coding repeat expansion disorder: five cases of spinocerebellar ataxia type 31 with nigrostriatal dopaminergic dysfunction

**DOI:** 10.1186/s40673-021-00134-4

**Published:** 2021-03-30

**Authors:** Ryohei Norioka, Keizo Sugaya, Aki Murayama, Tomoya Kawazoe, Shinsuke Tobisawa, Akihiro Kawata, Kazushi Takahashi

**Affiliations:** Department of Neurology, Tokyo Metropolitan Neurological Hospital, 2-6-1 Musashidai, Fuchu, Tokyo, 183-0042 Japan

**Keywords:** Spinocerebellar ataxia type 31, Nigrostriatal dopaminergic dysfunction, Midbrain atrophy, Parkinsonism, Magnetic resonance imaging planimetry, Non-coding repeat expansion disorder

## Abstract

**Background:**

Spinocerebellar ataxia type 31 (SCA31) is caused by non-coding pentanucleotide repeat expansions in the *BEAN1* gene. Clinically, SCA31 is characterized by late adult-onset, pure cerebellar ataxia. To explore the association between parkinsonism and SCA31, five patients with SCA31 with concomitant nigrostriatal dopaminergic dysfunction (NSDD) development, including three cases of L-DOPA responsive parkinsonism, were analyzed.

**Methods:**

To assess regional brain atrophy, cross-sectional and longitudinal imaging analyses were retrospectively performed using magnetic resonance imaging (MRI) planimetry. The midbrain-to-pons (M/P) area ratio and cerebellar area were measured on midsagittal T1-weighted MRI in five patients with SCA31 with concomitant NSDD (NSDD(+)), 14 patients with SCA31 without NSDD (NSDD(−)), 32 patients with Parkinson’s disease (PD), and 15 patients with progressive supranuclear palsy (PSP). Longitudinal changes in the M/P area ratio were assessed by serial MRI of NSDD(+) (*n* = 5) and NSDD(−) (*n* = 9).

**Results:**

The clinical characteristics assessed in the five patients with NSDD were as follows: the mean age at NSDD onset (72.0 ± 10.8 years), prominence of bradykinesia/akinesia (5/5), rigidity (4/5), tremor (2/5), dysautonomia (0/5), vertical gaze limitation (1/5), and abnormalities on ^123^I-ioflupane dopamine transporter scintigraphy (3/3) and 3-Tesla neuromelanin MRI (4/4). A clear reduction in the midbrain area and the M/P area ratio was observed in the NSDD(+) group (*p* < 0.05) while there was no significant difference in disease duration or in the pons area among the NSDD(+), NSDD(−), and PD groups. There was also a significant difference in the midbrain and pons area between NSDD(+) and PSP (*p* < 0.05). Thus, mild but significant midbrain atrophy was observed in NSDD(+). A faster rate of decline in the midbrain area and the M/P area ratio was evident in NSDD(+) (*p* < 0.05).

**Conclusion:**

The clinical characteristics of the five patients with SCA31 with concomitant NSDD, together with the topographical pattern of atrophy, were inconsistent with PD, PSP, and multiple system atrophy, suggesting that SCA31 may manifest NSDD in association with the pathomechanisms underlying SCA31.

**Supplementary Information:**

The online version contains supplementary material available at 10.1186/s40673-021-00134-4.

## Introduction

Spinocerebellar ataxia type 31 (SCA31) is an inherited neurodegenerative disorder characterized by slowly progressive, late adult-onset, pure cerebellar ataxia [1, 2]. SCA31 is caused by an insertion mutation of variable-length (2.5–3.8 kb) containing (TGGAA)n within the intron of the brain-expressed associated with NEDD4 1 (*BEAN1*) gene [3]. A few cases of SCA31 presenting with extracerebellar signs, including parkinsonism, postural tremor, dystonia, and spastic paraparesis, have been reported [4–6]. In Japan, genetic testing for SCA31 is usually performed in patients with pure cerebellar ataxia but yields a bias against extracerebellar manifestations of SCA31. Only a few patients with SCA31 have undergone neuropathological assessment, and there are no reports of autopsy cases of SCA31 presenting with extracerebellar signs except two cases in which dementia developed at the terminal stage [7, 8]. Therefore, the extracerebellar manifestations in patients with SCA31 are still largely unknown.

The present study analyzed five cases of SCA31 with nigrostriatal dopaminergic dysfunction (NSDD), defined as the presence of at least two of the three cardinal motor symptoms of Parkinson’s disease (PD), including resting tremor, rigidity, and bradykinesia/akinesia or one of the three cardinal motor symptoms plus an abnormal ^123^I-ioflupane dopamine transporter (DAT) scintigraphy finding. We aimed to describe the clinical characteristics of these cases and to explore the association between NSDD and SCA31. Cross-sectional and longitudinal imaging analyses were retrospectively conducted to assess regional brain atrophy in the midbrain and pons in the five patients with NSDD and control subjects.

## Patients and methods

### Patients and study design

**Fig. 1:**
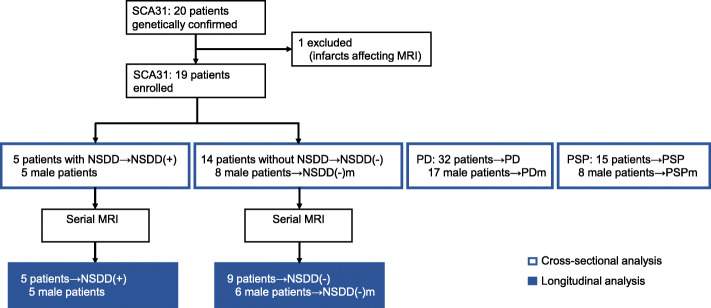
Schematic diagram of the study design. Number of patients included in the cross-sectional (□) and longitudinal (■) imaging analyses. All five patients with NSDD(+) were male. Therefore, for statistical comparison, the patients with NSDD(−), PD, and PSP were further divided into male patients with NSDD(−) (NSDD(−)m), male patients with PD (PDm), and male patients with PSP (PSPm). Serial MRI scans were done for all five patients with NSDD(+) and nine of 14 patients with NSDD(−), accounting for the difference in the number of patients with NSDD(−) between the cross-sectional and longitudinal analyses. NSDD, nigrostriatal dopaminergic dysfunction; NSDD(+), SCA31 with NSDD; NSDD(−), SCA31 without NSDD; PD, Parkinson’s disease; PSP, progressive supranuclear palsy; SCA31, spinocerebellar ataxia type 31

Between April 2010 and December 2019, 20 patients with cerebellar ataxia caused by a genetic mutation responsible for SCA31 were referred to our hospital. Of these, one patient was excluded due to having infarcts affecting the midline sagittal magnetic resonance imaging (MRI) assessments. In total, 19 patients with SCA31 underwent cross-sectional imaging analysis using MRI planimetry and were divided into the SCA31 with NSDD group (NSDD(+), *n* = 5) and the SCA31 without NSDD group (NSDD(−), *n* = 14). Serial brain MRI examinations and longitudinal imaging analysis were performed in all five patients with NSDD(+) and in nine of the 14 patients with NSDD(−). The study design and the number of patients included in the cross-sectional and longitudinal imaging analyses are summarized in Fig. 1.

Between January 2019 and July 2020, 39 patients with PD who fulfilled the Movement Disorder Society (MDS) clinical diagnostic criteria for ‘clinically established PD’ were referred to our hospital [9]. To exclude juvenile PD, three patients younger than 50 years at onset were excluded. PD is susceptible to clinical misdiagnosis, especially in its earlier stages. For this reason, four patients who had a disease duration of less than 3 years from onset were also excluded. In total, 32 patients with PD underwent cross-sectional imaging analysis.

Between January 2018 and July 2020, 15 patients with progressive supranuclear palsy (PSP) who fulfilled the MDS clinical diagnostic criteria for ‘probable PSP’ were referred to our hospital [10]. All these patients underwent cross-sectional imaging analysis.

### Clinical data

Clinical data were collected from the patients’ medical records. After obtaining informed consent, DAT scintigraphy and 3-Tesla (3-T) neuromelanin MRI were performed in three and four of the five patients with NSDD, respectively. DAT scintigraphy was not performed in one patient due to the lack of consent. DAT scintigraphy and 3-T neuromelanin MRI were not performed in another patient with NSDD due to the development of lung cancer. The 3-T neuromelanin MRI was performed in accordance with the previously described method [11].

### Gene analysis

After written informed consent was obtained, genomic DNA was extracted from peripheral-blood leukocytes and tested for SCA1, 2, 3, 6, 7, 8, 12, 17, and dentatorubral-pallidoluysian atrophy (DRPLA) using the previously described method [12]. SCA31 was diagnosed in patients showing both a single-nucleotide C → T substitution in the 5’UTR of the puratrophin-1 gene and a pentanucleotide insertion in the introns of the *TK2* and *BEAN1* genes [1–3]. To visualize the C → T substitution in the 5’UTR of the puratrophin-1 gene, the genomic DNA was amplified by PCR method (forward primer: 5′-CAGCGCGGTTCACACTGAGA-3′, reverse primer: 5′-GGCCCTTTCTGACAGGACTGA-3′), and the PCR product was digested by EcoN1. The patients with SCA31 had the C → T substitution in the mutant allele, which disrupted one EcoNI site and produced fragments of 268 and 92 bp [13]. Analysis of the insertion mutation was performed using the following method [14]. Briefly, genomic DNA 100 ng was mixed with 10 μM primer 0.75 μl (forward primer: 5′-ACTCCAACTGGGATGCAGTTTCTCAAT-3′, reverse primer: 5′-CTTTAGGGACCTGATTTCCTTCCTCCA-3′) in a total volume of 25 μl containing 12.5 μl 2X-PCR buffer for KODFX (TOYOBO), 400 μM dNTP, 5 μl H_2_O, and 0.5 μl KODFX (TOYOBO). The samples were denatured at 95 °C for 5 min, followed by 35 cycles at 95 °C for 20 s and at 68 °C for 8 min. The PCR products were run on 1.5% agarose gel.

### MRI planimetry

All the patients underwent brain MRI with a 3-T MRI scanner (750 GE). MRI-based planimetry on midsagittal T1-weighted MRI was performed to measure the area of the midbrain, pons, and cerebellum and to calculate the midbrain-to-pons (M/P) and the cerebellum-to-pons (C/P) area ratios. The pontomesencephalic junction was defined by a line between the superior pontine notch and the inferior border of the quadrigeminal plate [15]. The pontomedullary junction was defined by a line parallel to the first line at the level of the inferior pontine notch. Image analysis was done by blinded investigators using ImageJ software (version 1.52). First, a region of interest (ROI) was located in the area of the pontine tegmentum to derive the mean individual background signal and SD. Then, the ROIs were manually outlined as described above. To delineate the boundary of the ROIs, the area in each ROI was measured by a signal intensity value higher than the mean individual background signal minus 3SD (midbrain, pons) or the mean background signal minus 8SD (cerebellum). Two neurologists acting as independent raters blinded to the diagnosis analyzed the images.

### Cross-sectional imaging analysis

The area of the midbrain, pons, and cerebellum was measured in patients with SCA31, and the area of the midbrain and pons was measured in patients with PD and PSP. The latest MRI scan was used for imaging analysis in each patient who had repetitive MRI examinations.

### Longitudinal imaging analysis

Serial MRI scans were done for all five patients in the NSDD(+) group and nine of the 14 patients in the NSDD(−) group. Two MRI scans were removed for further study due to severe motion artifacts. In total, 40 MRI scans (15 for the NSDD(+) group and 25 for the NSDD(−) group) were used to measure the midbrain and pons area to calculate the M/P area ratio.

### Statistical analysis

Continuous variables were checked for normality and homogeneity of variance with Shapiro–Wilk’s and Levene’s tests. Normally distributed data were analyzed using Student’s t test or Welch test. The Mann-Whitney test was used when the variable was either ordinal or continuous but not normally distributed. The difference between the two groups was judged to be statistically significant if the *P* value was 0.05 or less. Linear regression analysis was performed to determine the effect of disease duration on regional brain atrophy. Normality and homoscedasticity of the residuals were checked in the linear regression analyses. Statistical analyses were performed using IBM SPSS Statistics 20.

## Results

### Clinical characteristics of patients with SCA31 complicated with NSDD

Of the 20 genetically confirmed cases of SCA31, NSDD developed in five patients, including four presenting with rigidity and bradykinesia/akinesia and one presenting with bradykinesia/akinesia with an abnormal DAT scintigraphy finding. According to the presence or absence of NSDD, the patients with SCA31 were divided into two groups (NSDD(+): SCA31 with NSDD, *n* = 5, NSDD(−): SCA31 without NSDD, *n* = 14). The clinical characteristics of cerebellar ataxia in SCA31, including slowly progressive, late adult-onset ataxia, eye movement abnormalities with saccade and horizontal gaze-evoked nystagmus, and prominent atrophy of the upper cerebellum, were equally observed in the NSDD(+) and NSDD(−) groups. The clinical and imaging features of parkinsonism and the related symptoms in the five patients in the NSDD(+) group (Table 1) were as follows: L-DOPA responsive parkinsonism (3/3), prominence of bradykinesia/akinesia (5/5), rigidity (4/5), tremor (2/5), dysautonomia (0/5), vertical gaze limitation (1/5), pons atrophy (0/5), putamen atrophy (0/5), and abnormal DAT scintigraphy (3/3), and 3-T neuromelanin MRI (4/4) findings. NSDD developed in all five patients with NSDD after the onset of cerebellar ataxia.

**Table 1:** Clinical characteristics of five patients with SCA31 complicated with NSDD

Patient	1	2	3	4	5
**Sex**	M	M	M	M	M
**Age at onset**	52	57	64	73	72
**Age at NSDD onset**	59	60	84	74	83
**Age at MRI**	63	61	85	77	83
**Family history**	+	+	+	–	+
**L-DOPA response**	+	+	+	NE	NE
**Symptoms**					
**Cerebellar ataxia**	+	+	+	+	+
**Rigidity**	+	+	+	–	+
**Bradykinesia/akinesia**	+	+	+	+	+
**Tremor**	+	–	–	–	+
**Vertical gaze palsy**	–	–	+	–	–
**Increased DTR**	+	+	+	–	+
**Dysautonomia**	–	–	–	–	–
**Brain MRI findings**					
**Cerebellar atrophy**	+	+	+	+	+
**MCP atrophy**	–	–	–	–	–
**Putamen atrophy**	–	–	–	–	–
**Hot cross bun sign**	–	–	–	–	–
**Neuromelanin MRI**					
**SN neuromelanin signal**	↓	↓	↓	↓	NE
**LC neuromelanin signal**	↓	↓	↓	→	NE
**RI findings**					
**DAT scintigraphy** **SBR (Right/Left)**	0.44/0.04	3.20/3.39	NE	3.08/2.71	NE

*Abbreviations*: *DAT* 123I-ioflupane dopamine transporter, *DTR* Deep tendon reflex, *LC* Locus coeruleus, *MCP* Middle cerebellar peduncle, *NE* Not examined, *NSDD* Nigrostriatal dopaminergic dysfunction, *SBR* Specific binding ratio, *SCA31* Spinocerebellar ataxia type 31, *SN* Substantia nigra

### Cross-sectional imaging analysis in patients with SCA31 complicated with NSDD

**Fig. 2:**
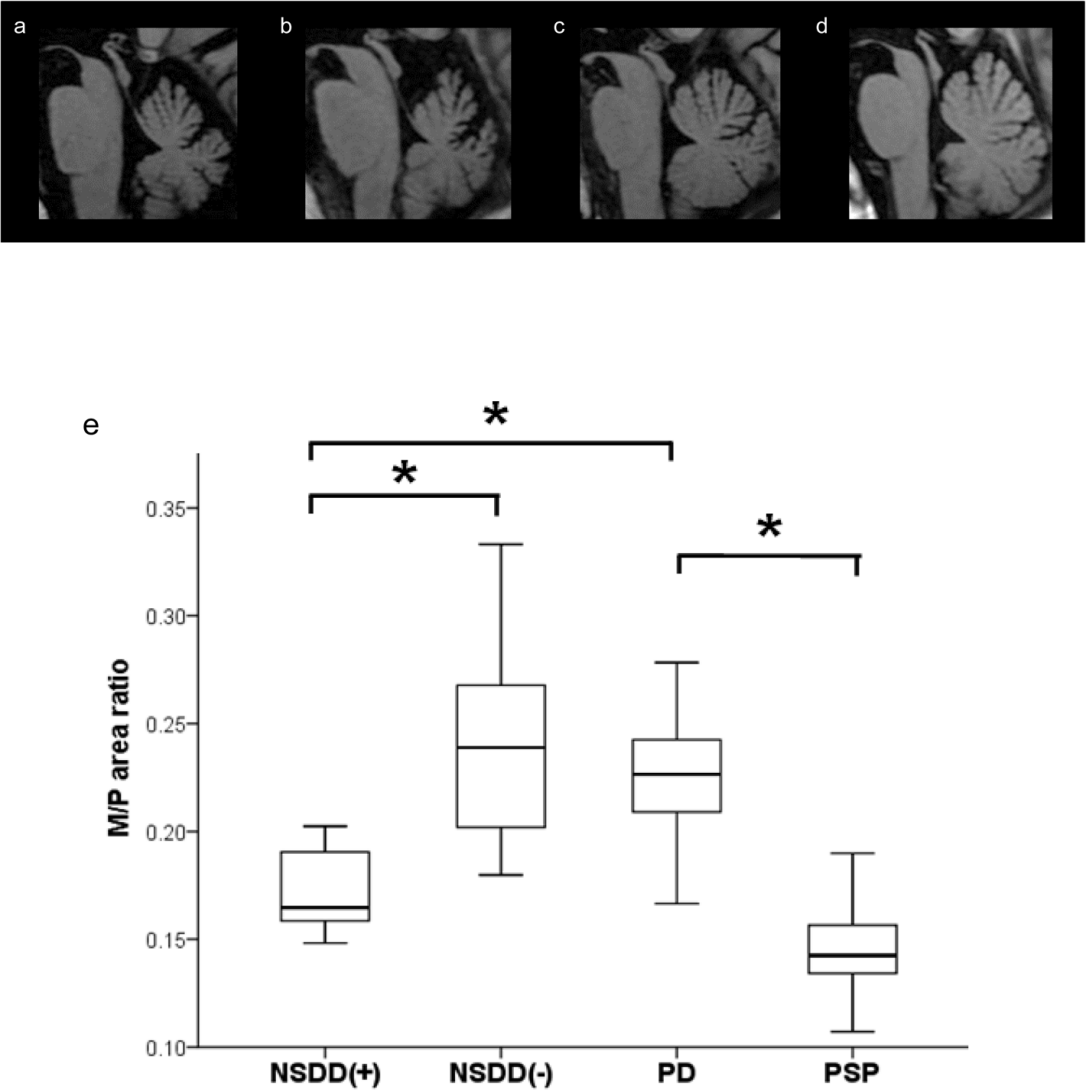
Midbrain atrophy in a representative case of NSDD and the results of M/P area ratio. Midsagittal T1-weighted MRI of a representative case from the NSDD(+) (**a**), NSDD(−) (**b**), PD (**c**), and PSP (**d**). **e** Cross-sectional imaging analysis of the M/P area ratio showing a clear difference between the NSDD(+) and NSDD(−) groups (*p* = 0.005), and between the NSDD(+) and PD groups (*p* = 0.001). In line with previous studies, the M/P area ratio showed a clear difference between the PD and PSP groups (*p* < 0.001). *Significant difference (*p* < 0.05). M/P, midbrain-to-pons; NSDD, nigrostriatal dopaminergic dysfunction; NSDD(+), SCA31 with NSDD; NSDD(−), SCA31 without NSDD; PD, Parkinson’s disease; PSP, progressive supranuclear palsy; SCA31, spinocerebellar ataxia type 31

Table 2 summarizes the demographic features of the 19 patients with SCA31 with or without NSDD, the 32 patients with PD and the 15 patients with PSP and the results of the cross-sectional imaging analysis. First, to estimate the influence of disease duration on the midbrain area, pons, and cerebellum, linear regression analysis was used to assess the relationship between disease duration at MRI acquisition versus the M/P area ratio and the relationship between disease duration at MRI acquisition and the C/P area ratio in all 19 patients with SCA31 (Supplementary Fig. 1). As expected, there was a significant effect of disease duration on the C/P area ratio (coefficient of determination, *R*^2^ = 0.305, *p* = 0.014). However, there was no significant effect of disease duration on the M/P area ratio (*R*^2^ = 0.157, *p* = 0.093), although there was a weak, linear relationship between the variables. All five patients with NSDD were male; thus, NSDD(−) was subdivided into male patients without NSDD (*n* = 8, NSDD(−)m). An apparent reduction in the midbrain area and the M/P area ratio was observed in NSDD(+) (*p* < 0.05) while there was no statically significant difference in disease duration at MRI acquisition or in the C/P area ratio among the NSDD(+), NSDD(−), and NSDD(−)m groups (Table 2). Furthermore, the NSDD(+) findings were compared to the findings in the 32 patients with PD and the 15 patients with PSP. For statistical comparison, these patients were further divided into male patients with PD (PDm) and male patients with PSP (PSPm). There was a clear difference in the midbrain area and the M/P area ratio between the NSDD(+) and PD groups and between the NSDD(+) and PDm groups (*p* < 0.05) (Table 2). In line with previous studies, the PSP group showed a severe reduction in the midbrain and pons area than the PD group [15–18]. There was also a clear difference in the midbrain and pons area between the NSDD(+) and PSP groups and between the NSDD(+) and PSPm groups (*p* < 0.05) (Table 2). These results suggested mild but significant midbrain atrophy in patients with SCA31 with concomitant NSDD. Figure 2 shows midbrain tegmentum atrophy in a representative case of NSDD(+) and the result of the comparison of the M/P area ratio in these groups. Image analysis by an independent investigator confirmed these results.

**Table 2:** Demographic features and results of cross-sectional imaging analysis using MRI planimetry

	NSDD(+)	NSDD(−)	NSDD(−)m	PD	PDm	PSP	PSPm
**Number**	5	14	8	32	17	15	8
**M**: **F ratio**	5: 0	8: 6	8: 0	17: 15	17: 0	8: 7	8: 0
**Age at disease onset**	63.6 ± 8.2 (52–73)	57.6 ± 7.8 (40–70) *p* = 0.191	54.6 ± 8.0 (40–65) *p* = 0.101	66.0 ± 6.2 (53–79) *p* = 0.462	67.0 ± 5.2 (55–75) *p* = 0.302	72.1 ± 8.2 (60–87) *p* = 0.074	67.9 ± 7.5 (60–85) *p* = 0.396
**Age at MRI**	74.0 ± 11.3 (61–85)	68.4 ± 13.5 (44–87) *p* = 0.415	65.3 ± 14.9 (44–87) *p* = 0.285	74.4 ± 6.0 (61–83) *p* = 0.936	75.7 ± 5.3 (66–83) *p* = 0.758	76.7 ± 7.7 (62–89) *p* = 0.546	72.8 ± 7.8 (62–88) *p* = 0.817
**Disease duration at MRI (year)**	10.4 ± 6.8 (4–21)	10.7 ± 8.2 (2–30) *p* = 0.889	10.6 ± 9.5 (2–30) *p* = 0.711	8.4 ± 3.8 (3–18) *p* = 0.562	8.7 ± 3.5 (3–17) *p* = 0.664	4.7 ± 3.1 (1–11) *p* = 0.028^a^	4.9 ± 3.3 (1–10) *p* = 0.047^a^
**Midbrain area (mm**^**2**^**)**	88.8 ± 15.2 (71.9–105.2)	122.2 ± 24.7 (82.7–172.6) *p* = 0.012^a^	122.0 ± 30.6 (82.7–172.6) *p* = 0.048^a^	113.3 ± 17.1 (72.1–147.3) *p* = 0.005^a^	114.1 ± 15.6 (88.7–141.7) *p* = 0.005^a^	67.6 ± 14.8 (40.9–96.3) *p* = 0.013^a^	62.8 ± 10.9 (40.9–73.8) *p* = 0.004^a^
**Pons area (mm**^**2**^**)**	512.5 ± 46.0 (455.2–566.9)	500.9 ± 43.8 (408.0–572.9) *p* = 0.620	514.7 ± 41.8 (459.8–572.9) *p* = 0.931	508.5 ± 63.1 (370.5–676.4) *p* = 0.890	535.6 ± 66.7 (418.0–676.4) *p* = 0.481	446.2 ± 46.8 (354.3–510.4) *p* = 0.013^a^	458.0 ± 41.6 (381.7–510.4) *p* = 0.049^a^
**Cerebellum area (mm**^**2**^**)**	622.7 ± 96.8 (501.8–732.0)	628.8 ± 102.2 (449.0–812.8) *p* = 0.909	649.9 ± 92.6 (510.3–812.8) *p* = 0.623				
**M/P area ratio**	0.173 ± 0.023 (0.148–0.202)	0.244 ± 0.047 (0.180–0.333) *p* = 0.005^a^	0.235 ± 0.049 (0.180–0.333) *p* = 0.022^a^	0.224 ± 0.031 (0.146–0.296) *p* = 0.001^a^	0.215 ± 0.027 (0.146–0.251) *p* = 0.006^a^	0.151 ± 0.026 (0.107–0.210) *p* = 0.111	0.137 ± 0.017 (0.107–0.157) *p* = 0.007^a^
**C/P area ratio**	1.214 ± 0.142 (1.034–1.395)	1.255 ± 0.176 (0.927–1.591) *p* = 0.639	1.278 ± 0.174 (1.119–1.613) *p* = 0.502				

Data are expressed as the mean ± SD (range)

*Abbreviations***:** *C/P* Cerebellum-to-pons, *M/P* Midbrain-to-pons, *NSDD* Nigrostriatal dopaminergic dysfunction, *NSDD(+)* SCA31 with NSDD, *NSDD(-)* SCA31 without, *NSDD* NSDD(-)m, male patients with SCA31 without NSDD, *PD* Parkinson’s disease, *PDm* Male patients with PD, *PSP* Progressive supranuclear palsy, *PSPm* Male patients with PSP, *SCA31* Spinocerebellar ataxia type 31

^a^ Significant difference between NSDD(+) and X, X = NSDD(−), NSDD(−)m, PD, PDm, PSP, PSPm

### Longitudinal imaging analysis in patients with SCA31 complicated with NSDD

**Fig. 3:**
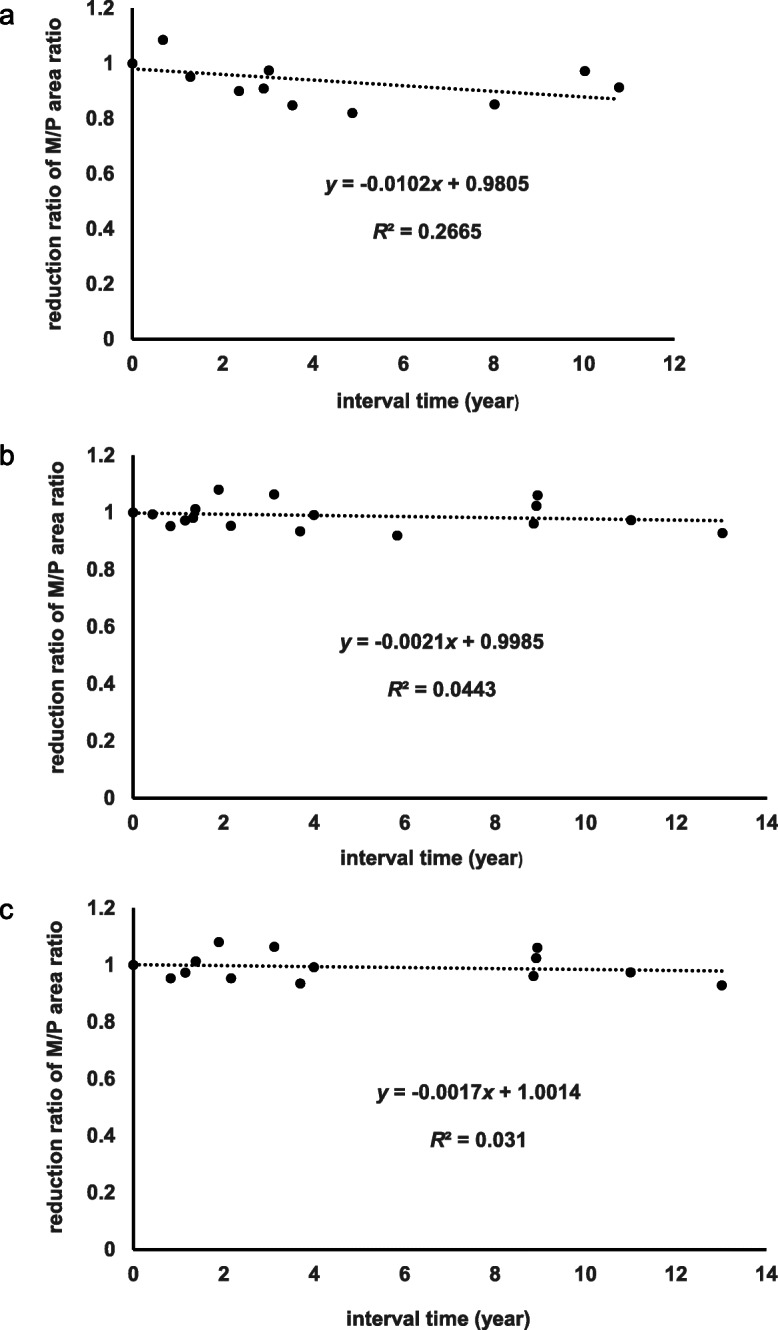
Linear regression analyses of the reduction ratio of the M/P area ratio over time. Longitudinal changes in the M/P area ratio were calculated by serial brain MRI obtained from the NSDD(+) (*n* = 5) (**a**) and NSDD(−) (*n* = 9) (**b**) groups. Male patients in the NSDD(−) group were placed into the male patients with NSDD(−) group (*n* = 6, NSDD(−)m) (**c**). *x* represents the time interval (year) from the day of the first MRI examination to the second or later MRI examination in each patient. The dependent variable *y* represents the reduction ratio of the M/P area ratio. The M/P area ratio derived from the first MRI examination was defined as 1, and the M/P area ratio derived from the second or later MRI was divided by the M/P area ratio derived from the first MRI in each patient. M/P, midbrain-to-pons; NSDD, nigrostriatal dopaminergic dysfunction; NSDD(+), SCA31 with NSDD; NSDD(−), SCA31 without NSDD; NSDD(−)m, male patients with SCA31 without NSDD; SCA31, spinocerebellar ataxia type 31

A previous, longitudinal study reported the regional rates of atrophy of 0.3%/year in the midbrain and 0.2%/year in the pons in healthy adult subjects [19]. From these data, the rate of reduction in the M/P area ratio was estimated to be 0.001/year (M/P area ratio at time 0 = 1). Thus, the M/P area ratio may be useful for minimizing the effect of age on midbrain atrophy. Longitudinal changes in the M/P area ratio were calculated using serial brain MRI obtained from patients with NSDD(+) (*n* = 5) and NSDD(−) (*n* = 9). The NSDD(−) group was further divided into male patients without NSDD (*n* = 6, NSDD(−)m). Table 3 summarizes the demographic features, average number of MRI examinations, and the mean interval between the MRI and the results of longitudinal imaging analyses in the three groups. To normalize the individual difference, the reduction ratio of the M/P area ratio was calculated as follows: the M/P area ratio derived from the first MRI examination was defined as 1, and the M/P area ratio derived from the second or later MRI was divided by the M/P area ratio derived from the first MRI in each patient. Liner regression of the reduction ratio of the M/P area ratio over time in the NSDD(+) group showed a significant effect of disease duration (*R*^2^ = 0.267, *p* = 0.0488) (Fig. 3a). However, liner regression of the reduction ratio of the M/P area ratio over time in the NSDD(−) and NSDD(−)m groups showed no significant effect of disease duration (*R*^2^ = 0.044, *p* = 0.312 and *R*^2^ = 0.031, *p* = 0.471, respectively) (Fig. 3b, c). The difference in the linear regression slopes (Fig. 3) between the NSDD(+) and NSDD(−) groups and between the NSDD(+) and NSDD(−)m groups was also tested (Supplementary File 1) and the result confirmed a faster rate of decline in the M/P area ratio in the NSDD(+) group. There was a clear difference in the reduction ratio of the midbrain area and the M/P area ratio in the NSDD(+) group (*p* < 0.05) while no significant difference in the reduction ratio of the pons area or in the mean interval between the MRI was observed among the three groups (Table 3). Thus, a faster rate of decline in the midbrain area and the M/P area ratio was evident in the NSDD(+) group. These results suggested significant progression of midbrain atrophy in patient with SCA31 with concomitant NSDD.

**Table 3:** Demographic features and results of longitudinal imaging analysis using MRI planimetry

	NSDD(+)	NSDD(−)	NSDD(−)m
**Number**	5	9	6
**M: F ratio**	5: 0	6: 3	6: 0
**MRI number**	3.0 ± 0.7 (2–4)	2.8 ± 1.0 (2–5) *p* = 0.422	3.2 ± 1.0 (2–5) *p* = 0.916
**Age at MRI**	69.2 ± 9.5 (56–86)	69.1 ± 10.3 (53–87) *p* = 0.970	69.3 ± 10.4 (53–87) *p* = 0.976
**Disease duration at MRI (year)**	6.5 ± 5.4 (1–21)	10.0 ± 8.5 (1–30) *p* = 0.175	11.4 ± 9.1 (1–30) *p* = 0.087
**Interval between MRI (year)**	3.2 ± 3.7 (0.0–10.8)	3.1 ± 4.0 (0.0–13.0) *p* = 0.842	3.6 ± 4.3 (0.0–13.0) *p* = 0.819
**Midbrain area (mm**^**2**^**)**	98.1 ± 14.5 (71.9–120.2)	114.1 ± 20.7 (82.7–149.8) *p* = 0.012^a^	110.5 ± 20.3 (82.7–149.5) *p* = 0.047^a^
**Pons area (mm**^**2**^**)**	526.0 ± 42.3 (455.2–604.9)	506.7 ± 34.0 (454.0–575.5), *p* = 0.121	507.6 ± 38.6 (454.0–575.5) *p* = 0.197
**M/P area ratio**	0.187 ± 0.026 (0.148–0.238)	0.224 ± 0.034 (0.176–0.306) *p* = 0.001^a^	0.216 ± 0.026 (0.176–0.260) *p* = 0.003^a^
**Reduction ratio of midbrain area**	0.898 ± 0.074 (0.773–0.995), *n* = 10	0.967 ± 0.046 (0.886–1.040), *n* = 16 *p* = 0.007^a^	0.968 ± 0.043 (0.893–1.040), *n* = 13 *p* = 0.009^a^
**Reduction ratio of pons area**	0.975 ± 0.062 (0.847–1.086), *n* = 10	0.979 ± 0.026 (0.943–1.033), *n* = 16 *p* = 0.859	0.976 ± 0.024 (0.943–1.017), *n* = 13 *p* = 0.985
**Reduction ratio of M/P area ratio**	0.922 ± 0.078 (0.820–1.085), *n* = 10	0.988 ± 0.049 (0.920–1.080), *n* = 16 *p* = 0.014^a^	0.993 ± 0.051 (0.928–1.080), *n* = 13 *p* = 0.016^a^

Data are expressed as the mean ± SD (range)

*Abbreviations*: *M/P* Midbrain-to-pons, *NSDD* Nigrostriatal dopaminergic dysfunction, *NSDD(+)* SCA31 with NSDD, *NSDD(−)* SCA31 without NSDD, *NSDD(−)m* Male patients with SCA31 without NSDD, *SCA31* Spinocerebellar ataxia type 31

^a^ Significant difference between NSDD(+) and NSDD(−) and between NSDD(+) and NSDD(−)m. In total, 40 MRI scans (15 for NSDD(+), 25 for NSDD(−), and 19 for NSDD(−)m were used to measure the midbrain and pons area to calculate the M/P area ratio

## Discussion

The M/P area ratio as measured on the midline sagittal images is reportedly a simple and reliable metric for distinguishing PSP from PD and multiple system atrophy (MSA) [15–18]. A statistically significant decrease in the midbrain area and the M/P area ratio was found in patients with SCA31 with concomitant NSDD (Table 2, Fig. 2). Compared to the PSP findings, the midbrain atrophy was mild, and no pontine atrophy was evident. Further observation of the progression of midbrain atrophy in these patients suggested that a relationship exists between midbrain atrophy and NSDD pathogenicity. Midbrain atrophy was also reported in patients with vascular parkinsonism [20]. In the present study, none of the five patients with NSDD had a medical history of stroke or diffuse subcortical white matter ischemia. The clinical characteristics relevant to parkinsonism in the patients with NSDD, together with the topographical pattern of atrophy, were inconsistent with those of PD, PSP or MSA. Furthermore, genetic testing excluded the other types of SCA, including SCA1, 2, 3, 6, 7, 8, 12, 17, and DRPLA. Thus, the findings of the present study raised the possibility of an association between NSDD and the pathomechanisms underlying SCA31 despite only a single-case report describing an association between SCA31 and parkinsonism being published to date [4].

Importantly, compared to a typical patient with PD, the SCA31 patients with NSDD in the present study showed relative prominence of bradykinesia in comparison with mild or absent rigidity and reduced spontaneous activity, including speech and voluntary movements. These features may lead to an underestimation of NSDD in patients with SCA31. Sever nigral degeneration without parkinsonism has been observed in disorders with cerebellar involvement, including SCA2 and SCA3 [21, 22], mitochondrial encephalopathy with *POLG* mutations [23], and MSA-C [24]. Furthermore, ipsilateral improvement of rigidity was reported in a patient with parkinsonism after a cerebellar stroke [25]. These findings suggest that cerebellar dysfunction can counteract the motor effects of nigrostriatal denervation and may ameliorate the clinical manifestations of parkinsonism, especially rigidity [23, 26]. Therefore, patients with SCA31 with NSDD development may manifest bradykinesia/akinesia without apparent parkinsonism (defined by the clinical combination of bradykinesia/akinesia, tremor at rest, and muscular rigidity). In such a context, NSDD may not be easy to recognize. Moreover, cerebellar dysfunction leads to slowing of movements, which is often experienced in patients with advanced ataxia. However, careful follow-up of these patients at regular intervals and detailed neurological examinations enabled the identification of subtle changes in muscle tonus and/or spontaneous activity that were difficult to explain by slowly progressive cerebellar ataxia. In such cases, DAT scintigraphy may now be used to elucidate nigrostriatal dopaminergic function. Clinically, cerebellar ataxia was the dominant manifestation in all five patients with NSDD and a main contributor of functional impairment in the activities of daily living, which were further vitiated by the concomitant NSDD.

SCA31 is a form of non-coding repeat expansion disorder that can be pathologically characterized by RNA foci formation and repeat-associated non-AUG translation [27, 28] The mechanism underlying non-coding repeat sequence-mediated neurotoxicity is still unclear. However, a previous study reported that above a pathological threshold repeat number, base pairing interactions drive liquid phase separation of RNA into membraneless gels as RNA foci [29]. The gelation of intracellular compartments may result in neurotoxicity by sequestering RNA binding proteins (RBPs) and inhibiting their normal function [29, 30]. A recent study found that ALS-linked RBP, TDP-43, and FUS, bound to and induced structural alteration of SCA31-associated UGGAA repeat expansion with the formation of RNA foci [31]. Interestingly, a mutation in the *TARDBP* gene encoding TDP-43 was identified as a cause of familial PD, presumably due to loss of TDP-43 function [32]. Thus, loss of function of RBPs binding specifically to UGGAA repeats within RNA foci can potentially explain the manifestation of NSDD in SCA31.

The present study has several limitations. Because the study was retrospective and enrolled a small number of subjects, estimating the actual incidence of NSDD in SCA31 and the gender difference in relation to the risk of developing NSDD was difficult. All five patients with NSDD were male, and SCA31 was characterized by late-adult onset. Therefore, gender and age effects on regional brain atrophy were carefully considered. To minimize these effects, the male patients with NSDD(−), PD or PSP were compared statistically and longitudinal changes in the M/P area ratio were assessed. Indeed, the M/P area ratio minimized the effect of age on regional brain atrophy (Fig. 3b, c). A growing number of diseases caused by non-coding repeat expansions are often associated with parkinsonism, including fragile X-associated tremor/ataxia syndrome (FXTAS) and neuronal intranuclear inclusion disease [33, 34]. Midbrain atrophy-related parkinsonism was reported in patients with FXTAS although its association with this disease remains elusive [35–37]. Further studies are required to clarify the association between NSDD and SCA31. This in turn will be helpful in understanding the pathomechanisms underlying SCA31 and parkinsonism associated with non-coding repeat expansion disorders.

## Conclusion

The present study revealed unique features of NSDD and shed light on its relationship to midbrain atrophy in patients with SCA 31, a disorder presumably presenting a pure cerebellar phenotype. The present study will hopefully expand our understanding of SCA31.

## Supplementary Information

**Additional file 1.**

**Additional file 2.**

## Data Availability

Data are available from the corresponding author on reasonable request.
