# Supplementary material for: Midbrain atrophy related to parkinsonism in a non-coding repeat expansion disorder: five cases of spinocerebellar ataxia type 31 with nigrostriatal dopaminergic dysfunction
Source: Cerebellum Ataxias. 2021 Mar 30;8:11. doi: 10.1186/s40673-021-00134-4 (PMC8010976; doi:10.1186/s40673-021-00134-4)
Supplement: Supplementary file 1 — Additional file 1. [file 40673_2021_134_MOESM1_ESM.pptx]

## Slide 1
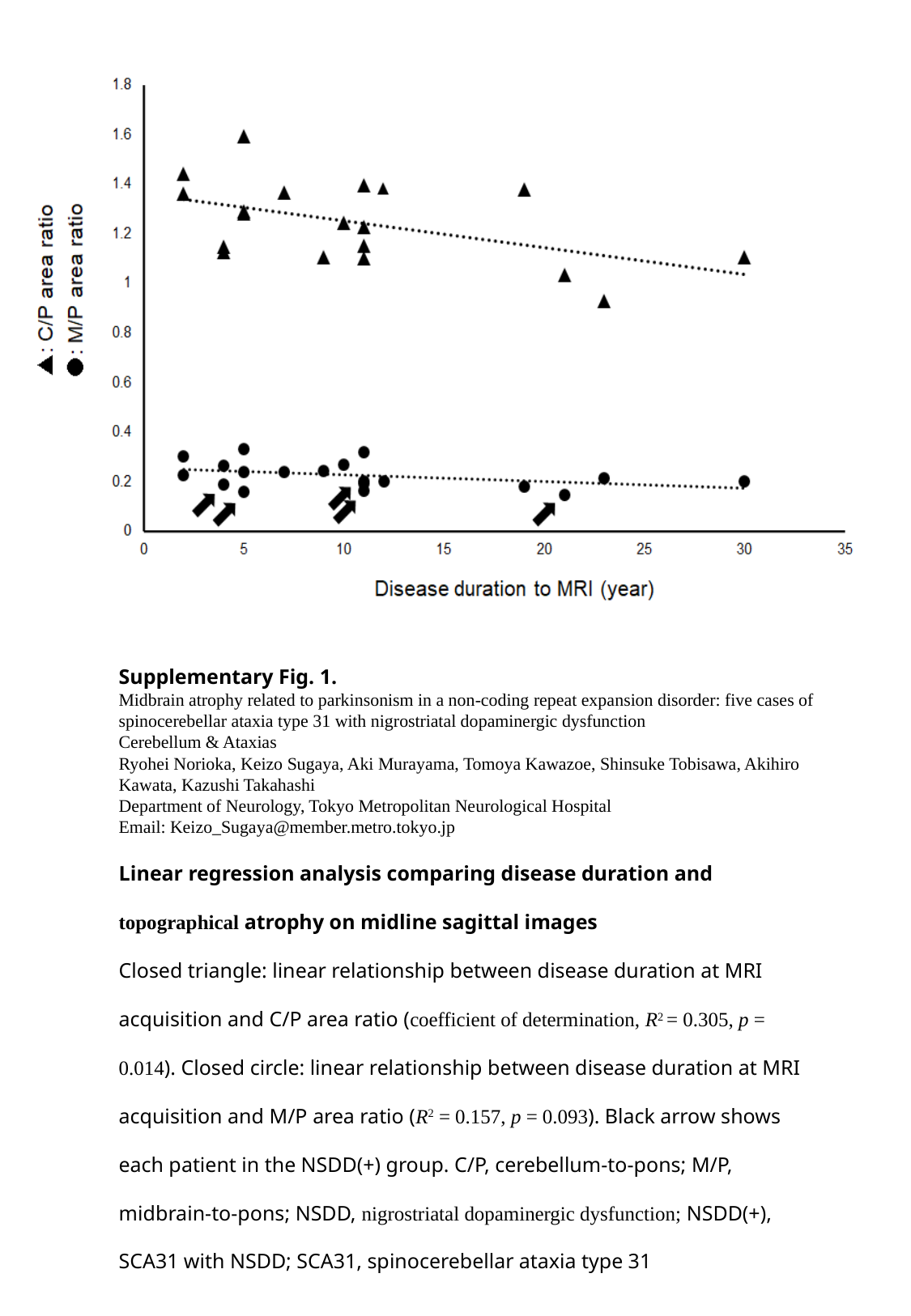

Supplementary Fig. 1.
Midbrain atrophy related to parkinsonism in a non-coding repeat expansion disorder: five cases of spinocerebellar ataxia type 31 with nigrostriatal dopaminergic dysfunction
Cerebellum & Ataxias
Ryohei Norioka, Keizo Sugaya, Aki Murayama, Tomoya Kawazoe, Shinsuke Tobisawa, Akihiro Kawata, Kazushi Takahashi
Department of Neurology, Tokyo Metropolitan Neurological Hospital
Email: Keizo_Sugaya@member.metro.tokyo.jp
Linear regression analysis comparing disease duration and topographical atrophy on midline sagittal images
Closed triangle: linear relationship between disease duration at MRI acquisition and C/P area ratio (coefficient of determination, R2 = 0.305, p = 0.014). Closed circle: linear relationship between disease duration at MRI acquisition and M/P area ratio (R2 = 0.157, p = 0.093). Black arrow shows each patient in the NSDD(+) group. C/P, cerebellum-to-pons; M/P, midbrain-to-pons; NSDD, nigrostriatal dopaminergic dysfunction; NSDD(+), SCA31 with NSDD; SCA31, spinocerebellar ataxia type 31
