# Supplementary material for: Midbrain atrophy related to parkinsonism in a non-coding repeat expansion disorder: five cases of spinocerebellar ataxia type 31 with nigrostriatal dopaminergic dysfunction
Source: Cerebellum Ataxias. 2021 Mar 30;8:11. doi: 10.1186/s40673-021-00134-4 (PMC8010976; doi:10.1186/s40673-021-00134-4)
Supplement: Supplementary file 2 — Additional file 2. [file 40673_2021_134_MOESM2_ESM.docx]

**Supplementary File 1**

Midbrain atrophy related to parkinsonism in a non-coding repeat expansion disorder: five cases of spinocerebellar ataxia type 31 with nigrostriatal dopaminergic dysfunction

Cerebellum & Ataxias

Ryohei Norioka, Keizo Sugaya, Aki Murayama, Tomoya Kawazoe, Shinsuke Tobisawa, Akihiro Kawata, Kazushi Takahashi

Department of Neurology, Tokyo Metropolitan Neurological Hospital

Email: Keizo_Sugaya@member.metro.tokyo.jp

The difference between two linear regression slopes was tested using the method described previously [1]. Briefly, according the following equations, the *t* value was calculated and compared to the critical *t* value with a significance level of 0.05. When the absolute value of the test statistic was greater than the critical *t* value, the null hypothesis was rejected.

Linear regression: (1) *y* = *a*_1_ + *b*_1_*x*

(2) *y* = *a*_2_ + *b*_2_*x*

Null hypothesis: *b*_1_ = *b*_2_

*s*_1_^2^ = S*_y_*_1_*_y_*_1_ (1 – *R*_1_^2^)

*s*_2_^2^ = S*_y_*_2_*_y_*_2_ (1 – *R*_2_^2^)

*s*^2^ = (*s*_1_ + *s*_2_)/(*n*_1_ + *n*_2_ – 4)

*t* = │*b*_1_ – *b*_2_│/*s* (1/ S*_x_*_1_*_x_*_1_ + 1/ S*_x_*_2_*_x_*_2_)^1/2^

where S*_x_*_1_*_x_*_1_, S*_y_*_1_*_y_*_1_, S*_y_*_2_*_y_*_2_, and S*_y_*_2_*_y_*_2_ represent the sum of the squared deviation of each variable in two groups. *R*_1_^2^ and *R*_2_^2^ represent the coefficient determination, and *n*_1_ and *n*_2_ represent the sample number per group.

The liner regression of the reduction ratio of the M/P area ratio over time (Fig. 3) was tested for the difference between two linear regression slopes.

NSDD(+): *y* = -0.0102*x* + 0.9805 (*R*^2^ = 0.2665)

NSDD(-): *y* = -0.0021*x* + 0.9985 (*R*^2^ = 0.0443)

NSDD(-)m: *y* = -0.0017*x* + 1.0014 (*R*^2^ = 0.031)

There was a significant difference in the linear regression slopes between the NSDD(+) and NSDD(-) groups (*t* = 1.832 > one-tailed *t* at 0.05 = 1.688). There was also a significant difference in the linear regression slopes between the NSDD(+) and NSDD(-)m groups (*t* = 1.761 > one-tailed *t* at 0.05 = 1.697).

Reference

[1] Dupont WD, Plummer WD Jr. Power and Sample Size Calculations for Studies Involving Linear Regression. Control Clin Trials. 1998;19(6):589-601.
